# Summary of the National Advisory Committee on Immunization (NACI) Seasonal Influenza Vaccine Statement for 2026–2027

**DOI:** 10.14745/ccdr.v52i09a01

**Published:** 2026-09-03

**Authors:** Angela Sinilaite, Winnie Siu, Kristin Klein, Jesse Papenburg

**Affiliations:** 1 Centre for Immunization Surveillance and Programs, Public Health Agency of Canada, Ottawa, ON; 2 School of Epidemiology and Public Health, Faculty of Medicine, University of Ottawa, Ottawa, ON; 3 NACI Influenza Working Group Chair; 4 Division of Preventive Medicine, Department of Medicine, University of Alberta, Edmonton, AB; 5 Division of Pediatric Infectious Diseases, Department of Pediatrics, Montréal Children’s Hospital, McGill University Health Centre, Montréal, QC; 6 Division of Microbiology, Department of Clinical Laboratory Medicine, Optilab Montréal, McGill University Health Centre, Montréal, QC; 7 Department of Epidemiology, Biostatistics, and Occupational Health, School of Population and Global Health, McGill University, Montréal, QC

**Keywords:** National Advisory Committee on Immunization, NACI, influenza, influenza vaccine, guidance, 2026–2027

## Abstract

**Background:**

The National Advisory Committee on Immunization (NACI) reviews evolving evidence on influenza immunization annually and updates vaccine recommendations. The NACI *Statement on seasonal influenza vaccines for 2026–2027* updates recommendations from the 2025–2026 season.

**Objective:**

To highlight new and updated information since the 2025–2026 statement and to summarize the 2026–2027 NACI seasonal influenza vaccine recommendations.

**Methods:**

The NACI Influenza Working Group applied the NACI evidence-based process to assess available evidence and formulate recommendations, which were evaluated and approved by NACI. Throughout the process, NACI’s peer-reviewed Ethics, Equity, Feasibility, and Acceptability (EEFA) framework, along with its evidence-informed tools (Ethics Integrated Filters, Equity Matrix, Feasibility Matrix, and Acceptability Matrix) were applied.

**Results:**

The overarching recommendation for seasonal influenza vaccination remains unchanged. Key updates for the 2025–2026 statement include: 1) integration of a new evidence review on optimal timing of seasonal influenza vaccination, including consideration of intraseasonal waning, and 2) integration of guidance on seasonal influenza vaccination in the context of avian influenza, with supporting resources.

**Conclusion:**

This guidance reaffirms NACI’s recommendation that influenza vaccine should be offered annually to anyone six months of age or older who does not have a contraindication to the vaccine.

## Introduction

Canada experiences annual seasonal influenza epidemics, primarily in the late fall and winter. The burden of influenza-associated illness and death varies each year due to factors such as circulating virus type, vaccine match to circulating strains and affected populations (1). Influenza activity in Canada is usually low in the late spring and summer, begins to increase over the fall, and peaks in the winter months. Multiple influenza strains typically circulate each season, but one strain often predominates. There are also often differences in the timing, intensity of influenza activity observed across regions in Canada.

Globally, approximately 3–5 million cases of severe influenza illness and 290,000 to 650,000 deaths from influenza occur annually (2). In Canada, prior to the COVID-19 pandemic (i.e., 2010–2011 to 2018–2019 influenza seasons), influenza caused approximately 15,000 hospitalizations annually, more than any other seasonal respiratory virus (3). Vaccination remains the most effective form of protection against influenza and its complications, underscoring the importance of annual vaccination programs.

Given the dynamic nature of influenza, the National Advisory Committee on Immunization (NACI) reassesses its guidance on influenza immunization on an annual basis and provides the Public Health Agency of Canada (PHAC) with recommendations on the use of authorized seasonal influenza vaccines. Annual updates reflect changes in influenza epidemiology, vaccine-related evidence (including safety, effectiveness, and programmatic considerations), immunization practices, and available products in Canada. The NACI Influenza Working Group leads the annual update of the NACI statement on seasonal influenza vaccines, which involves a thorough literature review and evaluation through deliberation at the scientific, clinical practice and population health levels. In May 2026, PHAC released updated NACI guidance on seasonal influenza vaccines for 2026–2027, based on current evidence and expert opinion.

This article provides a concise summary of NACI’s recommendations and supporting information for the 2026–2027 influenza season, with emphasis on new or updated information since the 2025–2026 statement on seasonal influenza vaccines. For detailed information, refer to NACI’s *Statement on seasonal influenza vaccines for 2026–2027* (4).

## Methods

In preparation for the *Statement on seasonal influenza vaccines for 2026–2027*, the NACI Influenza Working Group identified the need for evidence reviews on new topics and analyzed available evidence following NACI’s evidence-based process, which included developing updated recommendations as needed (5). Strength of NACI recommendations is defined in **Table A1** in the **Appendix**. NACI’s peer-reviewed framework and evidence-informed tools (including the Ethics Integrated Filters, Equity Matrix, Feasibility Matrix, and Acceptability Matrix) were applied to systematically assess issues of ethics, equity, feasibility and acceptability (6).

## Results

The overarching recommendation for seasonal influenza vaccination remains unchanged (see Summary of the NACI recommendations for the use of influenza vaccines for the 2026–2027 influenza season, below). The following sections summarize the key updates for the 2026–2027 statement.

### Intraseasonal waning of influenza vaccine effectiveness and optimal timing for seasonal influenza vaccination

The 2026–2027 seasonal influenza guidance includes an updated summary of the available evidence and key considerations regarding the optimal timing of seasonal influenza vaccine administration. This evidence review addresses the seasonality of influenza activity across Canada, intraseasonal waning of influenza vaccine-induced immunity and key programmatic considerations (7). While the current evidence is insufficient to establish optimal timing for specific populations, vaccination in early fall provides sustained protection for most of the season. Therefore, significant changes to the timing of existing annual influenza vaccination programs are neither warranted nor feasible at this time, and achieving high coverage remains the most critical determinant of program success.

### Seasonal influenza vaccination in the context of avian influenza

In February 2025, NACI published preliminary guidance on human vaccination against avian influenza in a non-pandemic context, offering a framework to inform Canadian provinces and territories’ decision-making on the use of human vaccines against avian influenza.

The seasonal influenza guidance has now been updated to align with this guidance on human vaccines against avian influenza, stating that although seasonal influenza vaccines do not protect against avian influenza A(H5N1) viruses, they may mitigate the severity of seasonal influenza and reduce the risk of co-infection with seasonal and avian influenza strains. Individuals whose occupational or recreational activities increase their risk of exposure to avian influenza A(H5N1) viruses are a group for whom influenza vaccination is particularly important.

For more information, refer to NACI’s *Rapid response: Preliminary guidance on human vaccination against avian influenza in a non-pandemic context as of December 2024* (8).

## Other updates

The seasonal statement for 2026–2027 also includes the following additional updates to the previous year’s statement: highlights of 2024–2025 influenza trends in Canada from new national surveillance data; vaccine products authorized and available for use in Canda for the coming season; a refined presentation of safety content to help ensure streamlined access to essential information on contraindications and precautions; and streamlined and updated appendices to improve usability and clarity.

### Summary of the NACI recommendations for the use of influenza vaccines for the 2026–2027 influenza season

NACI recommends that influenza vaccine should be offered annually to anyone six months of age and older who does not have a contraindication to the vaccine. Influenza vaccine should be prioritized for the groups for whom influenza vaccination is particularly important indicated in **List 1**. Refer to **Table 1** for the recommended influenza vaccine products and **Table 2** for the recommended dose and administration route according to each age group.

**Table 1:** Recommendations on choice of influenza vaccine type for individual and public health program-level decision making by age group

Recipient by age group	Vaccine types authorized^a^ and available for use	Recommendations on choice of influenza vaccine
6–23 months	IIV-Adj IIV-SD IIV-cc	• Any age-appropriate influenza vaccine should be used for infants and young children who do not have contraindications, noting the following considerations: o Influvac® (IIV3-SD) is authorized for use in children six months of age and older; however, additional NACI evidence review is required before recommending its use in children younger than three years. Updated guidance will be provided once this review is complete.
2–17 years^b^	IIV-SD IIV-cc LAIV	• Any age-appropriate influenza vaccine should be used for children and adolescents who do not have contraindications including those with chronic health conditions, noting the following considerations and exceptions: o Influvac® (IIV3-SD) is authorized for use in children six months of age and older; however, additional NACI evidence review is required before recommending its use in children younger than three years. Updated guidance will be provided once this review is complete. LAIV is contraindicated in children or adolescents with: ▪ Severe asthma (defined as currently on oral or high-dose inhaled corticosteroids) ▪ Active wheezing, or medically attended wheezing in the 7 days prior to vaccination ▪ Immune compromising conditions, with the exception of stable HIV infection, i.e., if the child is currently being treated with ART for at least four months and has adequate immune function ▪ Current receipt of long-term aspirin or aspirin-containing therapy ▪ Pregnancy
18–59 years	IIV-SD IIV-cc LAIV	• Any age-appropriate influenza vaccine should be used for adults 18 to 59 years of age who do not have contraindications, noting the following considerations and exceptions: o There is some evidence that IIV may provide better efficacy than LAIV in adults o LAIV is contraindicated in adults with: ▪ Severe asthma (defined as currently on oral or high-dose inhaled corticosteroids) ▪ Active wheezing, or medically attended wheezing in the 7 days prior to vaccination ▪ Immune compromising conditions ▪ Pregnancy o LAIV is not recommended for: ▪ Adults with any of the chronic health conditions identified in List 1 ▪ Healthcare workers
60–64 years	IIV-SD IIV-cc	Any age-appropriate influenza vaccine should be used for adults 60–64 years of age who do not have contraindications.
65 years and older^c^	IIV-Adj IIV-SD IIV-HD IIV-cc	IIV-HD, IIV-Adj, or RIV should preferentially be offered, when available, over IIV-SD or IIV-cc for adults 65 years of age and older without contraindications. If no preferred product is available, any available influenza vaccines authorized for this age group should be used.

Abbreviations: ART, antiretroviral therapy; IIV, inactivated influenza vaccine; IIV-Adj, adjuvanted inactivated influenza vaccine; IIV-cc, mammalian cell–culture-based inactivated influenza vaccine; IIV-HD, high-dose inactivated influenza vaccine; IIV-SD, standard-dose inactivated influenza vaccine; IIV4-SD, standard-dose quadrivalent inactivated influenza vaccine; LAIV, live attenuated influenza vaccine; RIV, recombinant influenza vaccine

^a^ Authorized products may or may not be marketed and available in Canada in a given season

^b^ Refer to Table 5 in NACI’s *Statement on seasonal influenza vaccines for 2026–2027* (4) for a summary of vaccine characteristics of LAIV compared with IIV in children 2–17 years of age

^c^ Refer to the NACI supplemental statement on influenza vaccination in adults 65 years of age and older (10) for rationale, supporting evidence appraisal and additional details on the evidence reviews that were conducted to support this recommendation

**Table 2:** Recommended dose and route of administration, by age, for influenza vaccine types authorized for the 2026–2027 influenza season

Age group	Influenza vaccine type (Route of administration)					Number of doses required
	IIV-SD^a^ (IM)	IIV-cc^b^ (IM)	IIV-Adj^c^ (IM)	IIV-HD^d^ (IM)	LAIV^e^ (Intranasal)	
6–23 months	0.5 mL^f^	0.5 mL	0.25 mL	N/A	N/A	1 or 2^g^
2–8 years	0.5 mL	0.5 mL	N/A	N/A	0.2 mL (0.1 mL per nostril)	1 or 2^g^
9–17 years	0.5 mL	0.5 mL	N/A	N/A	0.2 mL (0.1 mL per nostril)	1
18–59 years	0.5 mL	0.5 mL	N/A	N/A	0.2 mL (0.1 mL per nostril)	1
60–64 years	0.5 mL	0.5 mL	N/A	N/A	N/A	1
65 years and older	0.5 mL	0.5 mL	0.5 mL	0.7 mL	N/A	1

Abbreviations: IIV-Adj, adjuvanted inactivated influenza vaccine; IIV-cc, mammalian cell–culture-based inactivated influenza vaccine; IIV-HD, high-dose inactivated influenza vaccine; IIV-SD, standard-dose inactivated influenza vaccine; IM, intramuscular; LAIV, live attenuated influenza vaccine; N/A, not applicable

^a^ Fluzone® (6 months and older), Influvac® (6 months and older), Fluviral (6 months and older)

^b^ Flucelvax® Quad (6 months and older)

^c^ Fluad Pediatric^TM^ (6 to 23 months) or Fluad® (65 years and older). Although authorized, Fluad® Pediatric has not been available in Canada since 2019

^d^ Fluzone® High-Dose Quadrivalent (65 years and older)

^e^ FluMist® Quadrivalent (2 to 59 years)

^f^ Evidence suggests moderate improvement in antibody response in infants, without an increase in reactogenicity, with the use of full vaccine doses (0.5 mL) for unadjuvanted inactivated influenza vaccines. This moderate improvement in antibody response without an increase in reactogenicity is the basis for the full dose recommendation for unadjuvanted inactivated vaccine for all ages. For more information, refer to the *Statement on Seasonal Influenza Vaccine for 2011–2012* (11)

^g^ Children six months to less than nine years of age receiving seasonal influenza vaccine for the first time in their life should be given two doses of influenza vaccine, with a minimum interval of four weeks between doses. Children six months to less than nine years of age who have been vaccinated with one or more doses of seasonal influenza vaccine in the past should receive one dose of influenza vaccine per season thereafter

**List 1: Groups for whom influenza vaccination is particularly important**

**Table:** 

**People at high risk of influenza-related complications or hospitalization:** • All children 6 to 59 months of age • Adults and children with the following chronic health conditions^a^: o Cardiac or pulmonary disorders (including bronchopulmonary dysplasia, cystic fibrosis, and asthma) o Diabetes mellitus and other metabolic diseases o Cancer, immune compromising conditions (due to underlying disease, therapy, or both, such as solid organ transplant or hematopoietic stem cell transplant recipients) o Renal disease o Anemia or hemoglobinopathy o Neurologic or neurodevelopmental conditions (includes neuromuscular, neurovascular, neurodegenerative, neurodevelopmental conditions, and seizure disorders [and, for children, includes febrile seizures and isolated developmental delay], but excludes migraines and psychiatric conditions without neurological conditions) o Class 3 obesity (defined as body mass index of 40 kg/m^2^ and over) o Children 6 months to 18 years of age undergoing long-term treatment with acetylsalicylic acid, because of the potential increase of Reye’s syndrome associated with influenza • All pregnant women and pregnant individuals • All individuals of any age who are residents of nursing homes and other chronic care facilities • Adults 65 years of age and older • Individuals in or from First Nations, Inuit, or Métis communities as a result of intersecting determinants of health rooted in historic and ongoing colonization and systemic racism **People capable of transmitting influenza to those at high risk:** • Healthcare and other care providers in facilities and community settings who, through their activities, are capable of transmitting influenza to those at high risk • Household contacts, both adults and children, of individuals at high risk, whether or not the individual at high risk has been vaccinated: o Household contacts of individuals at high risk o Household contacts of infants less than 6 months of age, as these infants are at high risk but cannot receive influenza vaccine o Members of a household expecting a newborn during the influenza season • Those providing regular childcare to children 0 to 59 months of age, whether in or out of the home • Those who provide services within closed or relatively closed settings to people at high risk (e.g., crew on a cruise ship) **Others:** • People who provide essential community services • People whose occupational or recreational activities increase their risk of exposure to avian influenza A(H5N1)

^a^ Refer to immunization of persons with chronic diseases and immunization of immunocompromised persons Part 3 (9) of the *Canadian Immunization Guide* for additional information about vaccination of people with chronic diseases

## Conclusion

Annual influenza vaccination continues to be recommended by NACI for all individuals aged six months and older, noting product-specific age indications and contraindications. Adults 65 years of age and older should receive a high-dose or adjuvanted influenza vaccine, when available. Influenza vaccination is particularly important for groups identified in List 1.

## Appendix

**Table A1:** Strength of the National Advisory Committee on Immunization recommendations

Strength of NACI recommendation (Based on factors not isolated to strength of evidence, e.g., public health need)	Strong	Discretionary
Wording	“**should/should not** be offered”	“**may be** considered”
Rationale	Known/anticipated advantages outweigh known/anticipated disadvantages (“should”) OR known/anticipated disadvantages outweigh known/anticipated advantages (“should not”)	Known/anticipated advantages closely balanced with known/anticipated disadvantages OR uncertainty in the evidence of advantages and disadvantages exists
Implication	A strong recommendation applies to most populations/individuals and should be followed unless a clear and compelling rationale for an alternative approach is present	A discretionary recommendation may be considered for some populations/individuals in some circumstances Alternative approaches may be reasonable

Abbreviation: NACI, National Advisory Committee on Immunization
